# Type 1 diabetes in low and middle-income countries - Tanzania a streak of hope

**DOI:** 10.3389/fendo.2023.1043370

**Published:** 2023-03-24

**Authors:** Johnny Ludvigsson, Majaliwa Edna, Kaushik Ramaiya

**Affiliations:** ^1^ Crown Princess Victoria Children's Hospital and Division of Pediatrics, Department of Biomedical and Clinical Sciences, Linköping University, Linköping, Sweden; ^2^ Department of Pediatrics and Child Health, Muhimbili National Hospital, Dar es Salaam, Tanzania; ^3^ Hindu Mandal Hospital, Dar es Salaam, Tanzania; ^4^ Tanzanian Diabetes Association, Dar es Salaam, Tanzania

**Keywords:** low income, resources, type 1 diabetes, children, ketoacidosis, complications, Africa

## Abstract

**Introduction:**

In several of the Low and Middle Income countries , many patients with Type 1 diabetes (T1D) are most probably not diagnosed at all which may contribute to their low incidence. As an example of a country with low income and poor resources, we have chosen to study T1D in children/young people in Tanzania.

**Methods:**

Analyses of casebooks and statistics at several Tanzanian hospitals treating young patients with insulin dependent diabetes, usually Type 1 diabetes, and collection of information from different organisations such a Tanzanian Diabetes Association, Life for a Child, Changing Diabetes in Children and World Diabetes Foundation.

**Results:**

The incidence in several areas is low. However, a lot of data are often missing at studied clinics and therefore the incidence might be higher, and with increased awareness in recent years the number of patients has increased many-folds. Most patients present with typical symptoms and signs of T1D, and a high proportion with plausible ketoacidosis , although this proportion has decreased from about 90% to about 40% in recent decades. Many patients have poor blood glucose control, and complications often develop already after short diabetes duration. In recent years resources have increased, awareness has increased and diabetes clinics started where staff has got training.

**Conclusions:**

There are problems with diabetes care in Tanzania but several facts give hope for the future.

## Introduction

Type 1 diabetes (T1D) is one of the most common chronic diseases in children. It is supposed to be caused by an autoimmune process, with underlying cause unknown but believed to be a combination of genetic susceptibility (1) and environmental factors, such as beta cell stress, early nutrition, increased hygiene, or viral infections (2). Type 1 diabetes can occur at any age but is much more frequent in children and young adults.

Those diagnosed with type 1 diabetes need daily injections of insulin to survive and keep their blood glucose within acceptable levels. To sustain daily insulin injections, frequent daily blood glucose monitoring, education, and support to the child/young adult and their family is essential to ensure that those with type 1 diabetes get a reasonable quality of life with enough good blood glucose balance to avoid acute complications and prevent or delay late complications.

### Diagnosis and incidence of T1D

Diabetes can be classified using genetics and occurrence of autoantibodies which are present in 85-90% of the cases of classical T1D in the western world (3), and to some extent, also *via* C-peptide (4). However, the initial classification of T1D is usually based on the clinical presentation and family history (5). T1D is often diagnosed after a short period of symptoms such as fatigue, polyuria, polydipsia, and weight loss and the majority of patients in the western world (80–90%) have no family history of T1D. In Tanzania, a typical representative of Low or Middle-Income Countries (LMIC), children with diabetes are mostly regarded as T1D unless they have signs typical for Type 2 diabetes (T2D) like obesity and/or acanthosis nigricans. Even though African people have similar genetic susceptibility for T1D, HLA-DR3/DR4, as people in Europe (6, 7), idiopathic T1D (8) is more common in populations with African origin with no or reduced signs of autoimmunity (9, 10). Furthermore, T1D may include subtypes, which so far are not classified (11).

T1D accounts for approximately 10% of all patients with diabetes in the world (12). According to IDF Diabetes Atlas, 2021 (13), globally 1,211,900 children and adolescents younger than 20 years were estimated to have T1D, and it was estimated that around 149,500 children and adolescents below the age of 20 years are diagnosed each year. Numbers of children and adolescents under 20 years of age with type 1 diabetes in IDF AFRICA region (51,000) have more than doubled since 2019, probably mainly because of better diagnosis and availability of new data and the annual incidence is supposedto be ca19,000.

The incidence of Type 1 diabetes varies around the world. Scandinavia has the highest incidence (Finland ca 60 and Sweden ca 45 (14, 15)). There is an increasing knowledge about the incidence of T1D in Sub-Saharan Africa (13, 16, 17) and the incidence seems to increase (18).

In several LMIC, many patients with diabetes are most probably not diagnosed at all, which may contribute to the low incidence in these countries. A study 1993 on juvenile diabetes in Dar es Salaam, Tanzania, estimated the annual incidence to be 1.5/100,000 (19), while IDF 2013 estimated the incidence in children aged 0–14 years in Tanzania to be 0.9/100,000 per year (20). We did a study in recent years and found an annual incidence of 1.8–1.9/100,000 children, with an incidence peak at 10–14 years (21). However, a lot of data were missing at the studied clinics and therefore the incidence might be higher.

### Efforts to improve awareness and diagnosis of diabetes in Tanzania

The great majority of the patients in our study from Tanzania presented with typical signs and symptoms of T1D, and we estimated 83.7% to have presented with plausible ketoacidosis (DKA) (21). Furthermore, the frequency of misdiagnosis and subsequent deaths from DKA is not known but could be substantial. This should be compared with a much lower incidence of DKA at diagnosis in high-income countries (22).

Both in the health care and community an increased awareness of diabetes is needed. This is true in all countries, but especially so in LMIC. Efforts are done in Tanzania to improve the situation.

Tanzania Diabetes Association (TDA) was formed in 1985 as non-profit, non-Governmental organization (NGO) to unite the efforts of different people and stakeholders who were concerned with the care for people with diabetes in Tanzania. The purpose was not only to improve access to care for people living with diabetes in the country but also to put in strategies and initiatives to prevent and control diabetes.

TDA in collaboration with well-wishers, organizations, and institutions from within and outside Tanzania, and Government of Tanzania through the Ministry of Health (MoH) the President’s Office Regional Administration and Local Government (PORALG) has been able to establish network of diabetes/other non-communicable diseases (NCDs) clinics all over the country in tertiary and secondary public health facilities (Zonal, Regional, and District Hospitals) to improve access to care and quality of life of children, adolescents, and adults living with diabetes. At present, TDA is implementing further expansion into primary care facilities (Health Centers) all over the country.

**Figure 1:**
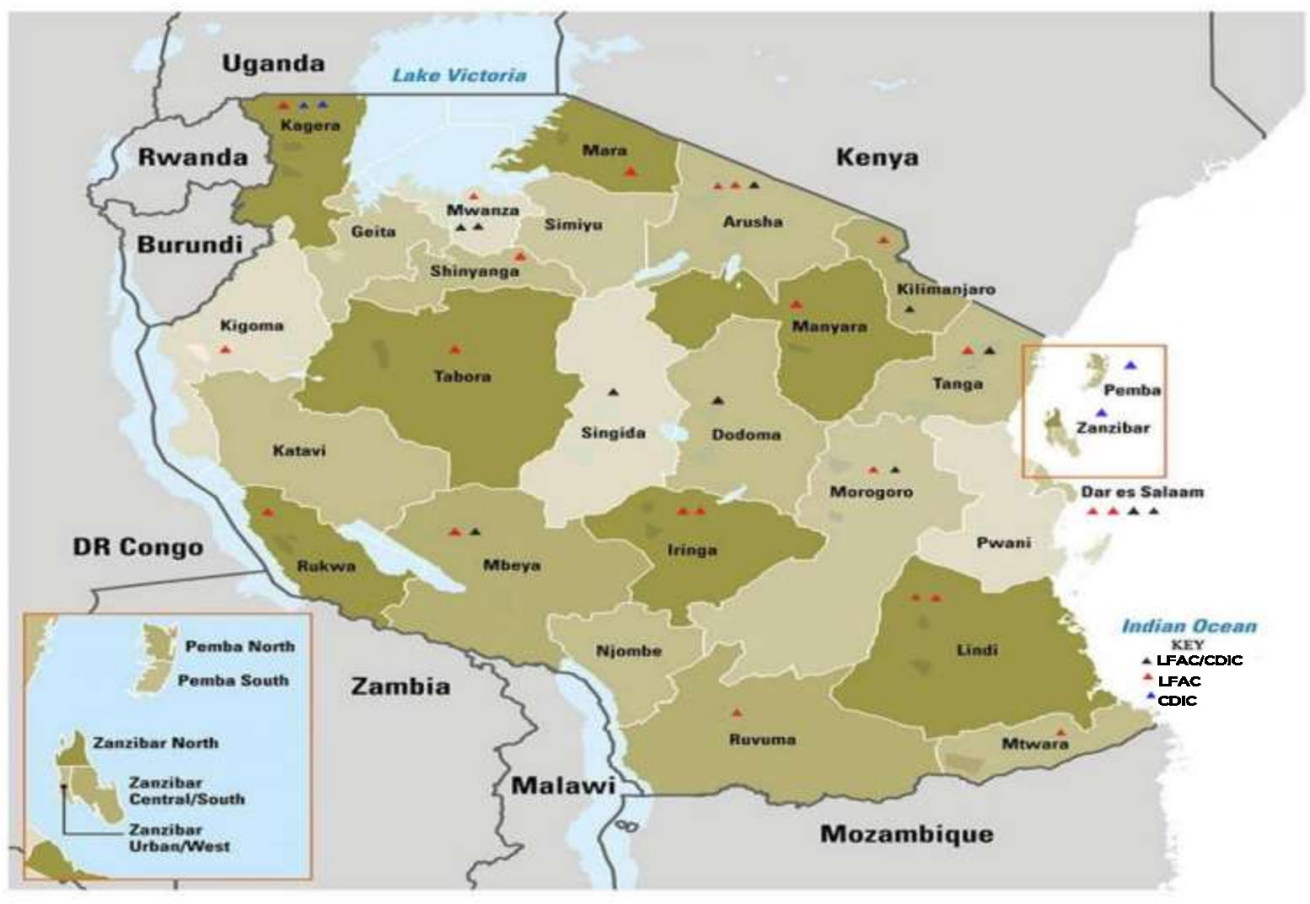
Geographical coverage of the type 1 diabetes clinic in Tanzania.

Since year 2005, TDA with support from funders such as Life for a Child Project (LFAC), Changing Diabetes in Children (CDiC) project and the World Diabetes Foundation (WDF) has continued to identify and enrol children and adolescents with type 1 diabetes at 38 clinics within the public-sector all-over Tanzania, including Zanzibar and are specific for improving type 1 diabetes services (**Figure 1**). Additional funding has been obtained recently to establish Type 1 diabetes clinics in the remaining regional hospitals in the country and also in lower-level hospitals/Health Centers where the number of children attending the clinic are more than 10.

**Figure 2:**
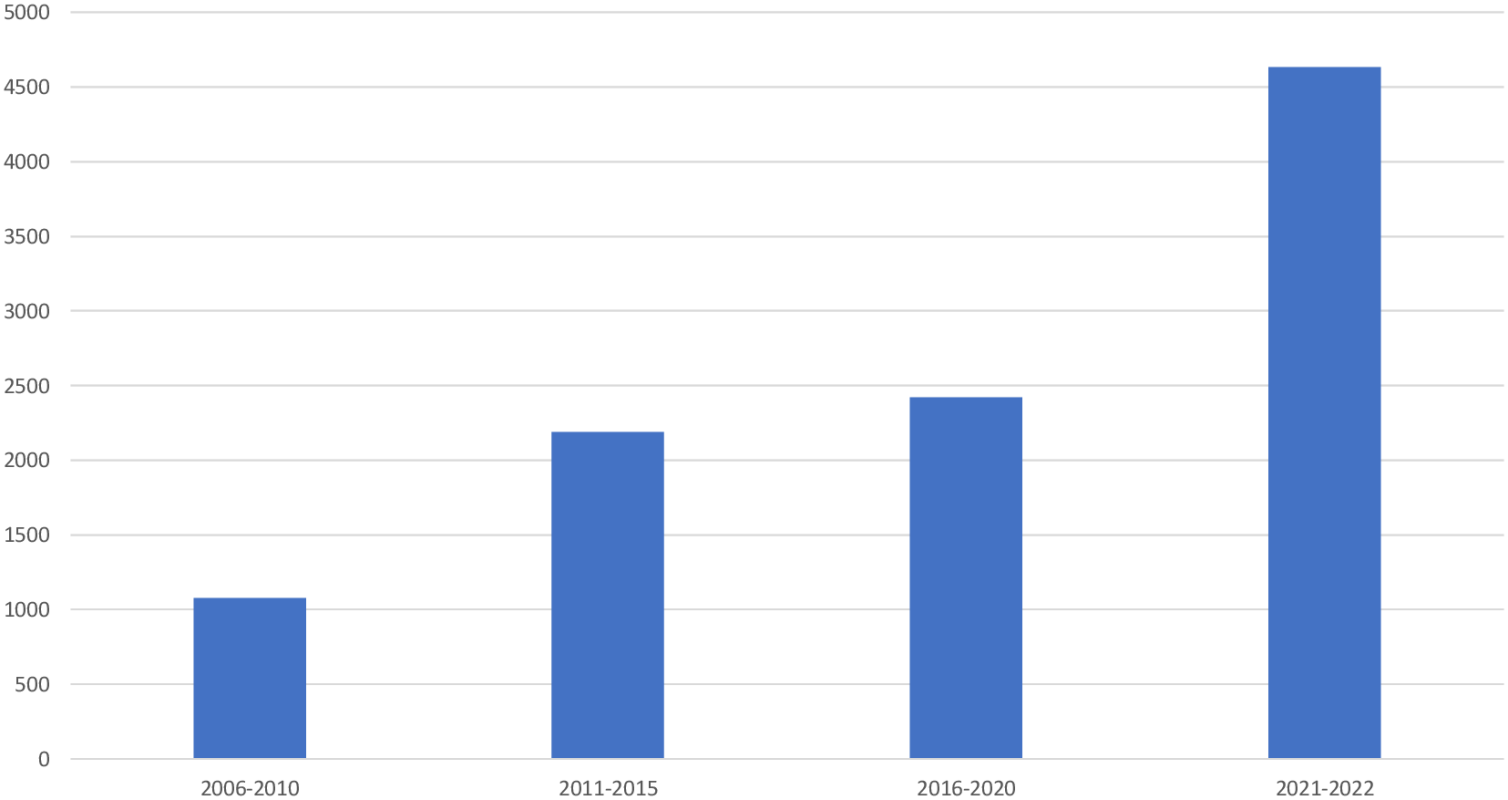
Total number of children with diabetes in Tanzania.

Thus, formally very much has been done, and progress has been seen, as shown by the increase in total number of children with diabetes (**Figure 2**) Still our own recent study (21), with investigations done both at national and regional centres, showed serious shortcomings. The great majority of patients lacked registered information on basal facts. One explanation to this may be that children with T1D did not come to their follow-ups at their diabetes clinic, another reason that HbA1c was not measured, or otherwise, that data were never registered. Only sporadic blood glucose values could be found, often registered as fasting blood glucose, although it was not clear if they were fasting. There were no registration or comments on home blood glucose values, which might have been used when HbA1c values were lacking. As a rule, even the most basal clinical information was lacking. A lot of missing data might, to some extent, be a reflection of limited resources, but even more an insufficient awareness and low priority among health care professionals.

In a majority of patients, HbA1c was never registered, but for those, we did find information the patients had overall poor blood glucose control, with calculated mean HbA1c 11.1% (98 mmol/mol) and most patients had HbA1c above 12.5% (113 mmol/mol). Others studies have got similar results (23). Even though progress is made in many countries, our findings from Tanzania are in agreement with those seen in other studies from Africa (24).

A poor blood glucose balance among children in Tanzania probably reflects the limitations of diabetes care, including lack of insulin supply (25, 26). When insulin is available, children in LMIC often get conventional insulin treatment in an inconsistent manner in contrast to developed countries where intensive diabetes treatment is offered. But lack of knowledge and motivation with inappropriate insulin doses also play an important role, poor monitoring practices missed doses of insulin, sometimes due to work place and school based stigma. Insulin supply with the program have improved but it is the “last mile” which is a challenge, i.e., frequent blood glucose testing, utilization of syringes, stigma at home/schools and insulin storage. Very hard work is necessary to improve awareness of diabetes both in the general population and in health care and this work is ongoing. The clinics in Tanzania are nowadays supplied on regular basis with insulin and other commodities such as glucometers, strips, lancets, and educational materials. Currently about 4000 youth living to type 1 diabetes have been registered with these clinics and they have access to these supplies at no cost. Thus, there is good grounds to improve care. Furthermore, health care workers from different facilities in the country have been trained in early diagnosis and management and are subjected to regular retraining. This is to ensure that they are updated on the latest science, knowledge, and skills required for the management of type 1 diabetes and they provide the highest quality of care to those in need. More than 350 healthcare workers have been trained on management of type 1 diabetes and additional numbers will be trained as TDA in partnership with MoH and PORALG, rolls out the National Type 1 diabetes and National NCD Program all over the country.

### Management of diabetes and prevention of complications

**Figure 3:**
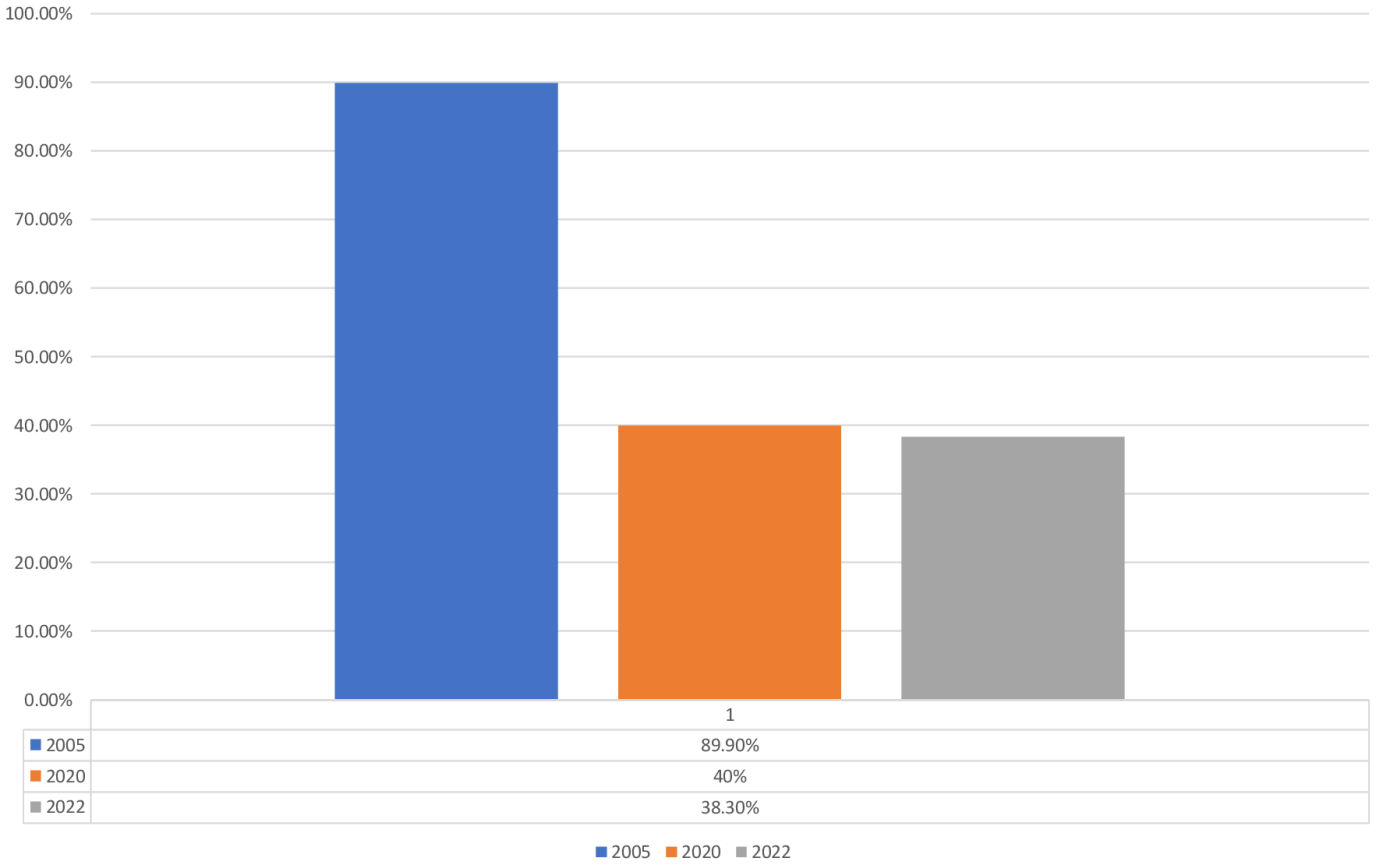
Trend of DKA in children and Youth with diabetes in Tanzania [Majaliwa, Edna., et al. “Diabetes care (2007);30.9: 2187-2192 (28); Kipasika, H., et al. “ Int J Diabetes Clin Res (2020); 7:126, Unpublished data].

Even when patients have got their access to insulin unfortunately structured management programs are often limited resulting in poor metabolic control, which we know causes both acute complications such as diabetes ketoacidosis, hypoglycemia, and chronic complications affecting the eyes, kidneys, nerves, and cardiovascular system. Complications are common in patients with T1D in LMIC and appear often after a rather short duration of the disease (27–33) in the worst case also increased mortality (23, 34, 35). Despite all the different challenges, the number of children diagnosed with DKA has been decreasing from about 90% to current 38% (**Figure 3**). But the true burden of type 1 diabetes in most LMIC is unknown. When we did a retrospective study analyzing medical recordings from 2010 – 2016 or 604 children and young adults with T1D who were recruited from five hospitals with pediatric diabetes clinics we had great difficulties to find information (28). The results showed a high prevalence of complications already after short duration of T1D, associated with poor metabolic control. Only 42.2% of the patients had registered HbA1c values, and among them, 36% had HbA1c >12.5%. There was high prevalence of retinopathy (21.5%) and neuropathy (29.4%) in spite of short mean duration of diabetes (6.2 ± 4.1 years). But as most data were missing the picture is unclear. HbA1C, plasma glucose, and complications were documented in less than half of the patient files. Thus, our results had to be interpreted with great caution, which is probably the same with other studies from LMIC.

### Efforts to improve treatment of diabetes in Tanzania

Thus, several studies have shown the typical and quite pronounced problems with diabetes care in Tanzania (21, 23, 27–29, 36). In many other LMIC, efforts are made to improve the care and there are several reports showing that such work is possible (24, 33, 37–39). A rather modest improvement of care can have great importance (40). In Tanzania, the Tanzania Diabetes Association (TDA) has been collaborating with several partners to train paediatric endocrinologists and support specialized diabetes courses for medical officers. These trained professionals are working within the public and private sectors to enhance their capacity the and be master trainers for other healthcare workers at different levels of health system in the country to improve diabetes care for all people in Tanzania. So far eight paediatric endocrinologists have been trained in Tanzania.

To ensure long term sustainability of supply chain for the above-mentioned 38 designated type 1 diabetes clinics, TDA together with MoH and PORALG have engaged with Medical Store Department (MSD) to ensure strengthening logistic and supply chain for insulin and other commodities, so that they reach clinics timely and access to care and delivery of services for children and adolescents with type 1 diabetes is uninterrupted.

TDA through community awareness campaigns and health education, has emphasized lifestyle modification and importance of healthy diet, physical exercise, tobacco control and avoidance of excessive alcohol intake as a critical components toward prevention and control of diabetes and other NCDs in the general community. TDA works in collaboration with other organizations and Tanzania NCD Alliance (TANCDA).

## Discussion

There is an improved survival of children with T1D in Africa, including Tanzania. The blood glucose control is of particular importance since it leads to improved quality of life and if left untreated results in premature morbidity and mortality.

Much improvement has been seen in survival, case finding, improvement in mortality, and record keeping. However, many children are still being missed as well as the missed diagnois resulting in too early mortality. Achievement of glycemic control has been poor for a long time. There has been lack of basic need for glycemic control which has been improving progressively. Therefore, more work is needed on managing and taking care of children and youth to be able to manage the glycemia as efforts are being done to improve the supply of insulin and monitoring tools, adherence is part of something to be worked on so more than just these supplies (25, 41, 42). In most studies the psychological parts of children, youth and parents is an aspect which is missing in the African context. Stigma to diabetes is still a hindrance factor of medication adherence, hence increasing the rate of poor glycemic control. Most of the children and youth would not want to show that they have diabetes, neither in schools or in public places nor even in marriages. Another hindrance to get good metabolic control in the limited resource setting is the lack of care givers involvement in the management of diabetes of their children. Furthermore, most of the diet is starch, which may sometimes increase the need for very high dose of insulin, necessitating children to use high insulin doses even when they are out of the puberty phase.

Diabetes awareness, still a gap on limited resources setting, is one of the contributing factor for the early complications, hence more interventions are needed. There is also a-lack of devices for frequent blood glucose monitoring in African countries. To add to that salt is the poor record keeping such that, more often than not, diagnosis and initial symptoms might be missing even in a hospital. Despite the fact that the level of DKA at presentation, children are still presenting very late to the health facility with symptoms of diabetes.

Limited resource setting needs to bridge the gap on family involvement to improve the diabetes care beyond insulin supply and blood glucose monitoring. There is need to move toward patient-centered diabetes education. As most of the setting, Africa lacks psychologists or diabetes educators in the teams, so there is need to equip whoever is available, be it doctor, nurse, or pediatrician, to deal with psychological questions and provide education.

Metabolic control has been unachievable for decades. Now our focus should move from insulin for survival to insulin together with education and psychological support for good control and a good quality of life both for parents and their families. Overcoming the “last mile” is the next challenge to obtain optimum control.

## Data availability statement

The original contributions presented in the study are included in the article/**Supplementary Material**. Further inquiries can be directed to the corresponding author.

## Ethics statement

The studies involving human participants were reviewed and approved by Ethics committé. Linköping university and DaresSalaam. Written informed consent to participate in this study was provided by the participants’ legal guardian/next of kin.

## Author contributions

JL had the idea, designed the project and wrote the first draft, ME and KR both contributed with further data, and worked with the manuscript. All authors contributed to the article and approved the submitted version.
