# Supplementary material for: Type 1 diabetes in low and middle-income countries - Tanzania a streak of hope
Source: Front Endocrinol (Lausanne). 2023 Mar 24;14:1043370. doi: 10.3389/fendo.2023.1043370 (PMC10080134; doi:10.3389/fendo.2023.1043370)
Supplement: Supplementary file 1 [file Table_1.docx]

Supplemental Table 1 LMIC

| **Complications** | **DKA AT DIAGNOSIS** | **DKA AFTER DIAGNOSIS** | **HYPOGLYCAEMIA** | **RETINOPATHY** | **NEPHROPATHY** | **NEUROPATHY** | **GLYCAEMIC CONTROL** |
| --- | --- | --- | --- | --- | --- | --- | --- |
| **YEAR** |  |  |  |  |  |  |  |
| **2005-2006** | **75%** | **89%** | **55%** | **22.6%** | **29.3%** |  | **12.5%** |
| **2009-2010** | **83%** | **68.8%** |  | **21%** |  | **29.4%** | **11%** |
| **2018-2020** | **33%** | **49%** |  | **10%** |  |  |  |

**Suppl Table 1. Trends of complications in type 1 diabetes : Tanzania**

Honesta K et al International Journal of diabetes 2021

S Najem et al journal of clin and translation endocrine 2020

Jasem D et Diabetes Research and Clinical Practice 2019

Majaliwa et Diabetes Care 2007
